# The role of Onodi cells in sphenoiditis: results of multiplanar reconstruction of computed tomography scanning^☆^^☆☆^

**DOI:** 10.1016/j.bjorl.2016.01.011

**Published:** 2016-04-20

**Authors:** Mehmet Senturk, Ibrahim Guler, Isa Azgin, Engin Umut Sakarya, Gultekin Ovet, Necat Alatas, Ismet Tolu, Omer Erdur

**Affiliations:** a Konya Education and Research Hospital, Department of Otolaryngology, Head, and Neck Surgery, Konya, Turkey; b Medical Faculty, Selçuk University, Department of Radiology, Konya, Turkey; c Konya Education and Research Hospital, Department of Radiology, Konya, Turkey; d Medical Faculty, Selçuk University, Department of Otolaryngology, Head, and Neck Surgery, Konya, Turkey

**Keywords:** Anatomic variation, Computed tomography, Onodi cell, Sphenoiditis, Variação anatômica, Tomografia computadorizada, Célula de Onodi, Esfenoidite

## Abstract

**Introduction:**

Onodi cells are the most posterior ethmoid air cells and extend superolateral to the sphenoid sinus. These cells are also intimately related with the sphenoid sinus, optic nerve, and carotid artery. Radiologic evaluation is mandatory to assess for anatomic variations before any treatment modalities related to the sphenoid sinus.

**Objective:**

To evaluate the effect of Onodi cells on the frequency of sphenoiditis.

**Methods:**

A retrospective analysis was performed in 618 adult patients who underwent high-resolution computed tomography between January 2013 and January 2015. The prevalence of Onodi cells and sphenoiditis was evaluated. Whether the presence of Onodi cells leads to an increase in the prevalence of sphenoiditis was investigated.

**Results:**

Onodi cell positivity was observed in 326 of 618 patients and its prevalence was found to be 52.7%. In the study group, 60.3% (*n* = 73) were ipsilaterally (*n* = 21) or bilaterally (*n* = 52) Onodi-positive, whereas 39.7% (*n* = 48) were Onodi-negative (*n* = 35) or only contralaterally Onodi-positive (*n* = 13). Of the control group, 48.3% (*n* = 240) were Onodi-positive and 51.7% (*n* = 257) were Onodi negative. The co-existence of Onodi cells ipsilaterally was observed to increase the identification of sphenoiditis 1.5-fold, and this finding was statistically significant (*p* < 0.05).

**Conclusion:**

The prevalence of sphenoiditis appears to be higher in patients with Onodi cells. However, it is not possible to state that Onodi cells are the single factor that causes this disease. Further studies are needed to investigate contributing factors related to sphenoiditis.

## Resumo

**Introdução:**

As células de Onodi são as células etmoidais mais posteriores, que se prolongam superolateralmente ao seio esfenoidal. Essas células também se encontram em íntima relação com o seio esfenoidal, o nervo óptico e a artéria carótida. Para análise de variações anatômicas antes da implementação de qualquer modalidade terapêutica relacionada ao seio esfenoidal, a avaliação radiológica é obrigatória,

**Objetivo:**

Nosso objetivo foi avaliar o papel das células de Onodi na frequência de esfenoidite.

**Método:**

Em nosso estudo, foi realizada uma análise retrospectiva em 618 pacientes adultos que se submeteram à tomografia computadorizada de alta resolução entre janeiro de 2013 e janeiro de 2015. Avaliamos a prevalência de células de Onodi e de esfenoidite. Investigamos se a presença de células de Onodi leva a um aumento na prevalência de esfenoidite.

**Resultados:**

A positividade para células de Onodi foi observada em 326 de 618 pacientes, e sua prevalência foi de 52,7%. No grupo de estudo, 60,3% (*n* = 73) eram CO-positivas: ipsilateral (*n* = 21) ou bilateralmente (*n* = 52); e 39,7% (*n* = 48) eram CO-negativas (*n* = 35) ou apenas contralateralmente CO-positivas (*n* = 13). No grupo de controle, 48,3% (*n* = 240) eram CO-positivas; e 51,7% (*n* = 257) eram CO-negativas. Observamos que a coexistência de CO ipsilateralmente aumentava em 1,5 vezes a associação com esfenoidite, e esse achado foi estatisticamente significante (*p* < 0,05).

**Conclusão:**

A prevalência de esfenoidite parece ser maior em pacientes com células de Onodi, mas não é possível afirmar que elas são isoladamente o fator causador desta doença. Novos estudos precisam ser realizados para uma investigação dos fatores contributivos relacionados à esfenoidite.

## Introduction

The Onodi cell (OC) is defined as the most posterior ethmoid cell, and may extend to the sphenoid sinus (SS) superiorly and laterally. The importance of these cells comes from their close relationship with the optic nerve (ON), SS, and hypophyseal fossa.^1^ Nomura et al.^2^ stated that OCs displace the SS downward, reducing its volume, and therefore could be associated with sphenoiditis. Ozturan et al.^3^ reported that the co-existence of the OC may alter the morphological changes in the floor and/or the lateral wall of the SS. In addition, it was mentioned that poor aeration and inefficient drainage of the OC lead to stasis of secretions, causing recurrent infections in mucoceles, optic neuritis, or optic neuropathies.^4^, ^5^, ^6^

Identification of OCs is possible using computed tomography (CT) scanning. It is necessary to examine all three dimensions (axial, coronal, and sagittal) meticulously to identify OCs. The accurate prevalence of OCs is not clear because CT scan studies of the prevalence of OCs in adults have produced varied results, ranging from 7% to 65%.^1^, ^3^, ^7^, ^8^, ^9^ Although there are studies on the prevalence of OCs in adult patients, it was not possible to find a study on the relationship between this anatomical variation and sphenoiditis. The only study found in PubMed was that conducted by Kim et al.^10^ with a child population, which reported that sphenoid sinusitis in children is not associated with the presence of OCs. Moreover, since development of the SS continues until the end of childhood,^11^, ^12^ a study on the relationship between the presence of OCs and sphenoiditis in adult patients will probably yield more reliable results than a study conducted with children.

In this study, the aim was to investigate whether the presence of OCs causes an increase in the frequency of sphenoiditis by analyzing thin-slice multiplanar (axial, coronal, and sagittal) reconstructed high-resolution computed tomography (HRCT) in adult patients with OCs, as well as gender and age profiles.

## Methods

Retrospectively, 618 adult patients who had received medical treatment for long-standing (>3 months) sino-nasal symptoms (nasal discharge, headache, cough, or nasal obstruction), had clinical findings (inflammatory findings were observed and confirmed with nasal endoscopic examination) of chronic sinus disease (not for allergic rhinitis or recurrent acute sinusitis), and had undergone paranasal sinus computed tomography (HRCT) in the Konya Education and Research Hospital between January 2013 and January 2015 were included in the study. Also, reviewing the patients’ records, those who had any history of trauma, nasal polyp, cystic fibrosis, asthma, immunosuppressive disease, malignancy, an opacification resembling a mass radiologically or a history of previous endoscopic sinus surgery, as well as patients with congenital malformations, were excluded from the study. The protocol of this study was approved by the institutional review board of the Medical Faculty of Meram, Necmettin Erbakan University, Konya.

In the Radiology Clinic, the routine CT imaging procedure steps were defined as follows: scans were performed with a 128-slice multidetector computed tomographic scanner (Ingenuity CT, Philips Healthcare, Andover, MA). Imaging parameters were as follows: Kv = 120; mA = 160; rotation time = 0.5 s; collimation = 64 × 0.625; FOV = 220 mm. The iterative reconstruction technique was employed to reduce radiation dose during scans. Axial images were recorded while the patient was in the supine position and the head was in a neutral position. The images covered the area from the apex of the frontal sinuses to the nasal maxillary process, parallel to the hard palate. Axial CT images were obtained with a section thickening of 0.625 mm, and these source data were used to obtain associated coronal and sagittal images with 0.9-mm slice thickness. Images were analyzed on a workstation (IntelliSpace Portal; Philips Healthcare – Andover, MA, United States). No patient underwent a new CT examination for this study. The retrospective analysis was performed using CT images recorded in the digital archive of the Radiology Clinic.

In patients who underwent an HRCT examination, the OC was defined as the most posterior ethmoidal air cell, extending superolaterally to the sphenoid sinus. After application of additional radiological criteria, such as CT scan quality and technical adequacy, by two independent observers (a radiologist and an otolaryngologist), 663 results of CT scans were examined. The OCs were determined by axial, coronal, and sagittal multiplanar HRCT scans. Identified OCs were divided as follows: (i) negative OC findings; (ii) right-sided OC findings; (iii) left-sided OC findings; (iv) bilateral OC findings. This study used the definition of sphenoiditis as the presence of mucosal thickening greater than 2 mm, as described by Gliklich and Metson.^13^ The sphenoiditis identified on CT were classified as follows: (a) negative sphenoiditis; (b) right-sided sphenoiditis; (c) left-sided sphenoiditis; (d) bilateral sphenoiditis. While the study group was consisted of sphenoiditis-positive patients, control group was consisted of sphenoiditis-negative patients. In the study group, Onodi-positive patients consisted of sphenoiditis-positive *plus* ipsilateral or bilateral OC-positive patients. Since the presence of unilateral Onodi cell is not expected to affect contralateral sphenoid sinus anatomically, it was considered that the presence of unilateral Onodi cells is not suitable to be in association with contralateral sphenoid sinusitis. Thus, the patients with sphenoiditis *plus* only contralateral Onodi cell positivity were also included into the OC-negative patients in study group. The frequencies of sphenoiditis in OC-positive and negative patients were calculated considering gender and age.

### Statistical methods

Univariate and multivariate logistic regression analyses were performed with forward logistic regression analysis to identify factors linked with OCs and sphenoiditis. OC, sphenoid sinusitis, gender, and ages were chosen as predictor variables. The categorized data were evaluated by the chi-squared test. Student's *t*-test for paired-samples was used to compare the same parameters with normal distribution. A *p*-value of 0.05 or less indicates a statistically significant difference. The analyses were performed using SPSS Statistics v.21, (IBM^®^ – New York, United States).

## Results

Six-hundred and eighteen patients meeting the study criteria were included; 353 were male (57.1%) and 265 were female (42.9%). The mean age was 36.4 years (range 18–87 years; median = 34 years). The mean age of females was 37.8 years, and the mean age of males was 35.4 years.

**Figure 1:**
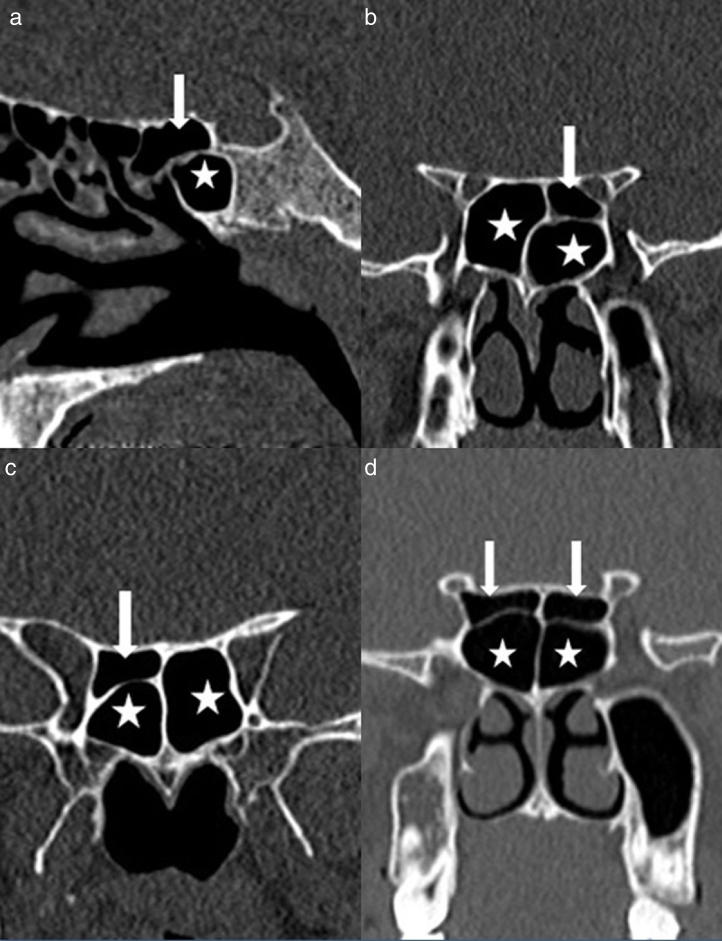
A coronal CT scan of the paranasal sinuses shows (a) sagittal image an Onodi cell; (b) coronal image of a left Onodi cell; (c) coronal image of a right Onodi cell; (d) coronal image of bilateral Onodi cells (arrow, Onodi cells; asterisk, sphenoid sinuses).

Onodi cell positivity was observed in 326 of 618 patients and its prevalence was found to be 52.7%. Of the 326 OC-positive patients, 28.8% (*n* = 94) were right-sided, 23.9% (*n* = 78) left-sided, and 47.3% (*n* = 154) bilateral (Fig. 1).

**Figure 2:**
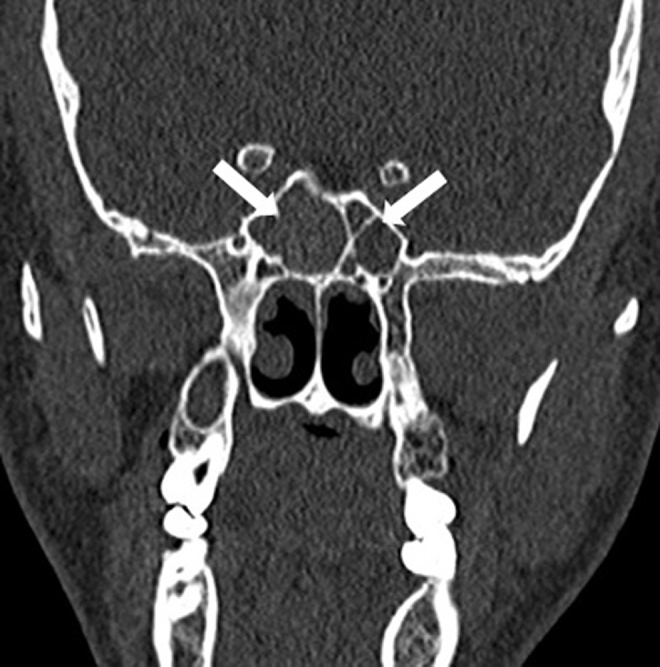
The CT scans of the paranasal sinuses shows bilateral sphenoiditis (arrows).

**Figure 3:**
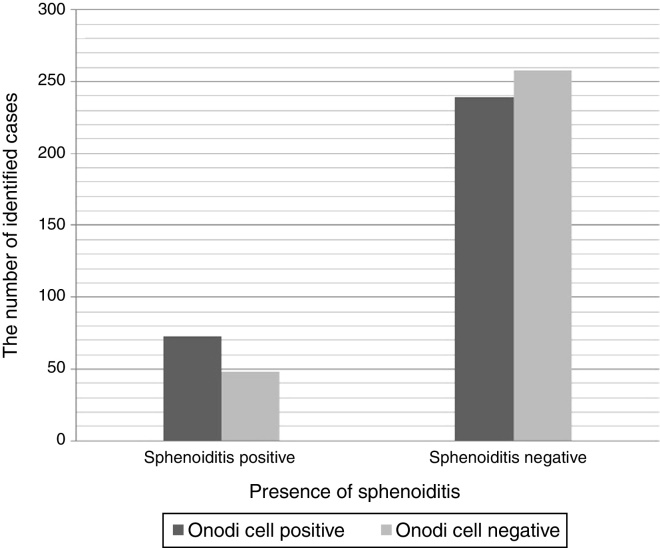
The graph shows that Onodi cell positivity causes a 1.5-fold increase in the number of cases with sphenoiditis (*p* = 0.018).

While 121 patients (19.6%) with sphenoiditis accepted as the study group, 497 patients (80.4%) without sphenoiditis accepted as the control group. Of the study group, 60.3% (*n* = 73) consisted of male patients and 39.7% (*n* = 48) were female patients. Sphenoiditis was significantly higher in males than in females (*p* < 0.05). Right-sided sphenoiditis was identified in 38% (*n* = 46), left-sided sphenoiditis in 31.4% (*n* = 38), and bilateral sphenoiditis in 30.6% (*n* = 37) (Fig. 2). In the study group, 13 patients who had only contralateral OC-positivity were accepted as OC-negative. Of the study group, 60.3% (*n* = 73) were ipsilateral (*n* = 21) or bilateral (*n* = 52) OC-positive, and 39.7% (*n* = 48) were OC-negative (*n* = 35) or only contralateral OC-positive (*n* = 13) (Table 1). The co-existence of OC ipsilaterally was observed to increase the identification of sphenoiditis 1.5-fold, and this finding was statistically significant (*p* < 0.05) (Fig. 3).

**Table 1:** Cross tabulation of sphenoiditis and Onodi cells.

Presence of Onodi cell (*n*)	Presence of sphenoiditis (*n*)			
	Right sphenoiditis (*n* = 46)	Left sphenoiditis (*n* = 38)	Bilateral sphenoiditis (*n* = 37)	Negative sphenoiditis (*n* = 497)
Right Onodi cell (*n* = 94)	12	5	8	69
Left Onodi cell (*n* = 78)	8	9	5	56
Bilateral Onodi cell (*n* = 153)	14	10	14	115
Negative Onodi cell (*n* = 293)	12	14	10	257

There were 280 (56.3%) male patients and 217 (43.7%) female patients in the control group. Of the control group, 48.3% (*n* = 240) were OC-positive, whereas 51.7% (*n* = 257) were OC-negative. Of the 240 OC-positive patients of the control group, right-sided OC was identified in 13.9% (*n* = 69) patients, left-sided OC in 11.3% (*n* = 56) patients, and bilateral OC in 23.1% (*n* = 115) patients.

## Discussion

Chronic sinus disease may impair the quality of life, and the SS, as well as all sinuses, may be affected by the chronic sinusitis disease processes. Endoscopic endonasal sinus surgery is currently accepted treatment modality for chronic sinusitis if medical treatment is insufficient.^14^, ^15^ In addition, small anatomical variations may be present around the paranasal sinuses. The OC is a sphenoethmoidal cell and is one of the cell variations around the SS. Săndulescu et al.^16^ suggested that important variations occur at the sphenoethmoidal junction, and most of these variations are related to the presence of the OC and intrasinusal protrusions of the ON. Ozturan et al.^3^ stated that OC pneumatization may reach and surround the ON in various extensions.

An accurate evaluation of these structures is possible with HRCT. The HRCT scan can clearly show the relationship between the OC and the sphenoid sinus. The multiplanar reconstruction technique has recently been developed as a new imaging technique in the field of CT.^17^ The reported studies regarding the prevalence of OCs vary greatly, and computed tomography (CT) scans suggest that prevalence is between 7% and 65%.^1^, ^3^, ^7^, ^8^, ^9^, ^18^ In cadaver studies, this prevalence was found to be 60% by Tanaviratananich et al.^19^ and 15% by Tan and Ong.^20^ In the present study, multiplanar (axial, coronal, sagittal) reconstructed HRCT scans and thin slices were used, and OCs were found in 52.7% of the patients. This finding was consistent with the literature.

Numerous studies reported that OCs have clinical significance for various reasons. When using endoscopy, the OC may easily be confused with the SS. Nomura et al.^2^ reported that the OC displaces the SS downward and reduces its volume, and so could be associated with sphenoiditis. In a CT study^1^ regarding the relationship between the OC and the sphenoid ostium (SO), it was found that the OC caused the vertical angles and distances from the SO to the OC become larger, which would result from the SO being displaced more inferiorly in the Onodi group, so it would be located farther from the superolateral position of the ON. Ozturan et al.^3^ stated that the coexistence of the OC may alter the morphological changes in the floor and/or the lateral wall of the SS. Chee et al.^4^ stated that poor aeration and drainage of the Onodi air cells lead to stasis of secretions and cause the patient to be prone to recurrent infections. The OC may be associated with mucoceles and optic neuritis because of these possible anatomic variations.^5^, ^6^

Analysis of the relationship between anatomical variations in paranasal sinuses and chronic rhinosinusitis on CT scans of 113 children found that OCs were not significantly correlated with sphenoid sinusitis.^10^ However, in that study, children were between 5 and 16 years of age, so development of pneumatization of the sphenoid sinus was not completed in all patients, and the OC was observed in only 11 patients. Additionally, the characteristics of sinusitis in children may be very different from those of adults. No studies have investigated the relationship between OC and sphenoiditis in adults. Regarding patients with sphenoiditis, 60.3% (*n* = 73) were ipsilateral or bilateral OC-positive patients and 39.7% (*n* = 48) were OC-negative or only contralateral OC-positive patients. The co-existence of OC was observed to increase the identification of sphenoiditis by 1.5-fold, which was statistically significant.

This study has some limitations: when the considering the developing the sinusitis in general, it is not possible to state that OC is the single factor that causes this disease. In this connection, as this study is a cross sectional study, even though it was observed that the presence of sphenoiditis was more prevalent in patients with OC, it is not possible to attribute causality among this study factor and the outcome. In patients with sphenoid sinusitis, other locational and dimensional features of OCs may be needed to be explored regarding this intimate relationship, such as degree of aeration and whether or not the drainage pathways of the sphenoid sinuses are corrupted. In addition, the definitive diagnosis of sinusitis can be established by sinus cavity cultures.^21^ However, in the case of sphenoiditis, it is very difficult to obtain sinus cavity culture sampling because anatomically reaching the sinus cavity is nearly impossible in outpatient conditions, except interventional conditions. To provide optimal conditions for diagnosis of sinusitis, the authors observed and confirmed the purulent secretion flowing down from the sinuses under nasal endoscopic examination. Further studies may be useful to establish exact nature of sphenoidal disease in the cases with co-existence of OC and sphenoiditis by means of culture sampling from the sphenoid sinus cavity during intervention.

## Conclusion

Although sphenoiditis was more frequently observed in patients with an OC in this study, it is not possible to state that the OC is the single factor that causes this disease. This study offers a new perspective regarding the relationship between the OC and sphenoiditis using multiplanar reconstructed thin-slice HRCT images, and further studies are needed to investigate contributing factors related to sphenoiditis.

## Conflicts of interest

The authors declare no conflicts of interest.
